# Case Report: Identification of microduplication in the chromosomal 2p16.1p15 region in an infant suffering from pulmonary arterial hypertension

**DOI:** 10.3389/fcvm.2023.1219480

**Published:** 2023-10-23

**Authors:** Xun Wang, Zeying Zhang, Wanyun Zuo, Dan Wang, Fan Yang, Qiming Liu, Yunbin Xiao

**Affiliations:** ^1^ Department of Cardiology, Hunan Children’s Hospital, Changsha, China; ^2^ Department of Cardiovascular Medicine, The Second Xiangya Hospital of Central South University, Changsha, China; ^3^ Xiangya School of Medicine, Central South University, Changsha, China

**Keywords:** pulmonary arterial hypertension, developmental delay, microduplication, copy number mutation, 2p16.1p15

## Abstract

This study reports the first case of a patient with chromosomal 2p16.1p15 microduplication syndrome complicated by pulmonary arterial hypertension (PAH). A female infant was admitted to the hospital suffering from dyskinesia and developmental delay, and conventional echocardiography revealed an atrial septal defect (ASD), which was not taken seriously or treated at that time. Two years later, preoperative right heart catheterization for ASD closure revealed a mean pulmonary artery pressure (mPAP) of 45 mmHg. The mPAP was reduced, and the condition was stabilized after drug therapy. A genomic copy number duplication (3×) of at least 2.58 Mb in the 2p16.1p15 region on the paternal chromosome was revealed. Multiple Online Mendelian Inheritance in Man (OMIM) genes are involved in this genomic region, such as *BCL11A*, *EHBP1*, *FAM161A*, *PEX13*, and *REL*. *EHBP1* promotes a molecular phenotypic transformation of pulmonary vascular endothelial cells and is thought to be involved in the rapidly developing PAH of this infant. Collectively, our findings contribute to the knowledge of the genes involved and the clinical manifestations of the 2p16.1p15 microduplication syndrome. Moreover, clinicians should be alert to the possibility of PAH and take early drug intervention when facing patients with 2p16.1p15 microduplications.

## Introduction

Pulmonary hypertension (PH) is a group of diseases characterized by a progressive increase of mean pulmonary arterial pressure (mPAP), with pulmonary vascular remodeling as the main pathological mechanism, which leads to right heart failure and eventually death. Pulmonary arterial hypertension (PAH) belongs to group 1 PH, and congenital cardiovascular disease is the most common cause of PH in children. Despite early detection and surgical repair of congenital cardiovascular defects, there is no better treatment for this type of PH. Moreover, it is considered that the underlying gene mutations or chromosomal aberrations are the major contributors to PH associated with pathological congenital cardiovascular disease.

Three case reports have documented 2p16.1p15 microduplication syndrome, and these cases have similar clinical manifestations, namely, developmental delay, intellectual disability, and congenital defects, but the length of the chromosomal microduplication region, the genes involved, and the number of genes differ (1–3). In addition to the developmental delay, this case also showed PH associated with a congenital defect of the heart and ocular lesions. Chromosomal alterations, especially duplication of certain segments, cause great genomic instability. Whole exome sequencing, karyotype analysis, copy number analysis, and single nucleotide polymorphism (SNP) analysis were performed to study this infant who suffered from PAH, developmental delay, movement disorders, and ocular lesions. A microduplication chromosomal abnormality in the 2p16.1p15 region was found. It is crucial to enrich the understanding of the genes involved and clinical manifestations of 2p16.1p15 microduplication syndrome.

## Case presentation

**Figure 1:**
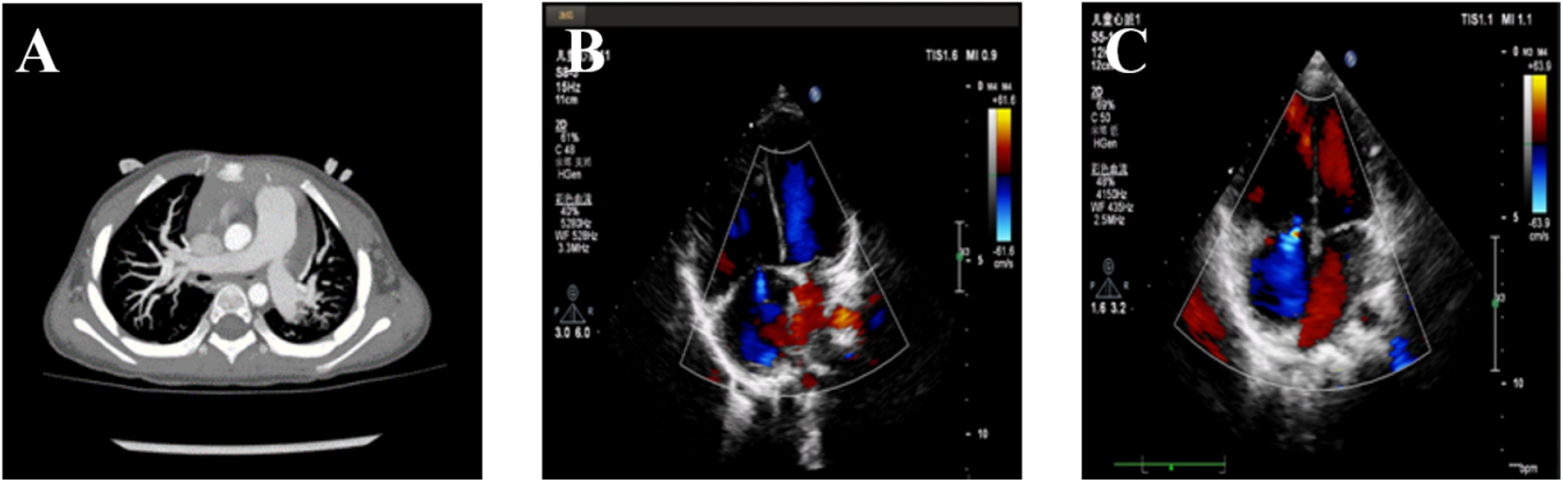
Pulmonary arterial hypertension of the patient. (**A**) The computerized tomography scan shows a dilated pulmonary artery. (**B**) The echocardiogram shows tricuspid regurgitation prior to treatment. (**C**) The echocardiogram shows tricuspid regurgitation after treatment.

A 2-year-old female infant was admitted to the Department of Cardiology of the Hunan Children's Hospital to undergo atrial septal defect (ASD) closure due to multiple echocardiographic examinations indicating an ASD (Figure 1). A preoperative echocardiogram revealed an ASD with mild tricuspid regurgitation. The ASD was carefully measured as 10 mm during the operation. The mPAP and pulmonary vascular resistance (PVR) were measured at 45 mmHg and 9.1 Wood units via right heart catheterization under transesophageal echocardiography guidance, respectively. The closure operation was canceled due to the infant having significant PAH. By puncturing the right femoral artery and inserting a right coronary catheter, we measure the blood pressure measured at various sites. The left ventricular pressure, ascending aortic pressure, and descending aortic pressure were 31, 48, and 48 mmHg, respectively, which ruled out pulmonary hypertension due to left heart-related disease. The oxygen saturations of the aorta, pulmonary artery, and superior vena cava were 100%, 72.3%, and 69.2%, respectively. PH crisis was considered, and the infant was given pure oxygen to prevent the possible occurrence of a PH crisis. The blood oxygen saturation of the infant was maintained above 90%, and the hemodynamics improved. There was a decrease in hemoglobin and transfusion of concentrated red blood cells used to improve anemia (Table 1).

**Table 1:** Changes in blood cell count and ratio in the patient.

Date	Leucocyte (10^9^/L)	Neutrophil ratio	Lymphocyte ratio	Hemoglobin (g/L)	Platelet (10^9^/L)
2 February 2019	9.99	0.365	0.533	113	359
8 April 2019	8.41	0.246	0.604	105	269
13 June 2019	8.16	0.332	0.531	98	328
13 March 2021	13.63	0.545	0.357	117	86
12 May 2021	13.85	0.346	0.573	128	101
16 June 2021	14.76	0.45	0.476	114	82
13 July 2021	11.68	0.407	0.503	117	81
16 July 2021	13.35	0.71	0.241	77	43
19 July 2021	13.59	0.45	0.433	81	112
18 August 2021	5.95	0.305	0.622	98	70
21 December 2021	11.43	0.481	0.432	124	85
1 March 2022	9.27	0.462	0.464	110	97
10 June 2022	6.65	0.299	0.574	101	31
8 July 2022	12.57	0.625	0.339	102	312
18 December 2022	6.18	0.5	0.414	97	78

After her condition improved and her vital signs stabilized, bosentan (22 mg/time/12 h) and sildenafil (5 mg/time/8 h) were applied to lower the mPAP. After her condition improved and she was discharged from the hospital, she continued to take bosentan and sildenafil orally. Because the follow-up test of the blood routine showed that thrombocytopenia (Table 1) and pulmonary arterial pressure did not decrease significantly, she was rehospitalized in June 2022 in our hospital. Gamma globulin was used to increase the number of platelets for 3 days. Other therapeutic drugs included bosentan tablets (25 mg/time/12 h) and sildenafil (5 mg/time/8 h) to treat PAH. Because the mPAP was estimated to be increased by echocardiogram, sildenafil was changed to tadalafil (7 mg/time/day) and re-examination of echocardiogram showed that the pulmonary artery pressure decreased.

When the infant was 3 months old, she was admitted to the Hunan Children's Hospital for treatment due to poor head-up in the prone position. The doctor observed that her limbs moved slowly, and her pronunciation was less. Through physical examination, it was found that she could not turn over in the supine position while the upper limbs did not shift with the fulcrum on the face when trying to raise her head in the prone position. She had hypotonia in the limbs with grade II muscle tone in both upper limbs and grade I muscle tone in both lower limbs. Retinal examination revealed focal ridge-like changes and hemorrhages in the temporal periphery of both eyes. Brain MRI showed enlargement and deformation of the bilateral ventricle and third ventricle. After rehabilitation treatment, her motor ability has improved. Her parents and relatives did not have corresponding clinical manifestations or similar medical history.

**Figure 2:**
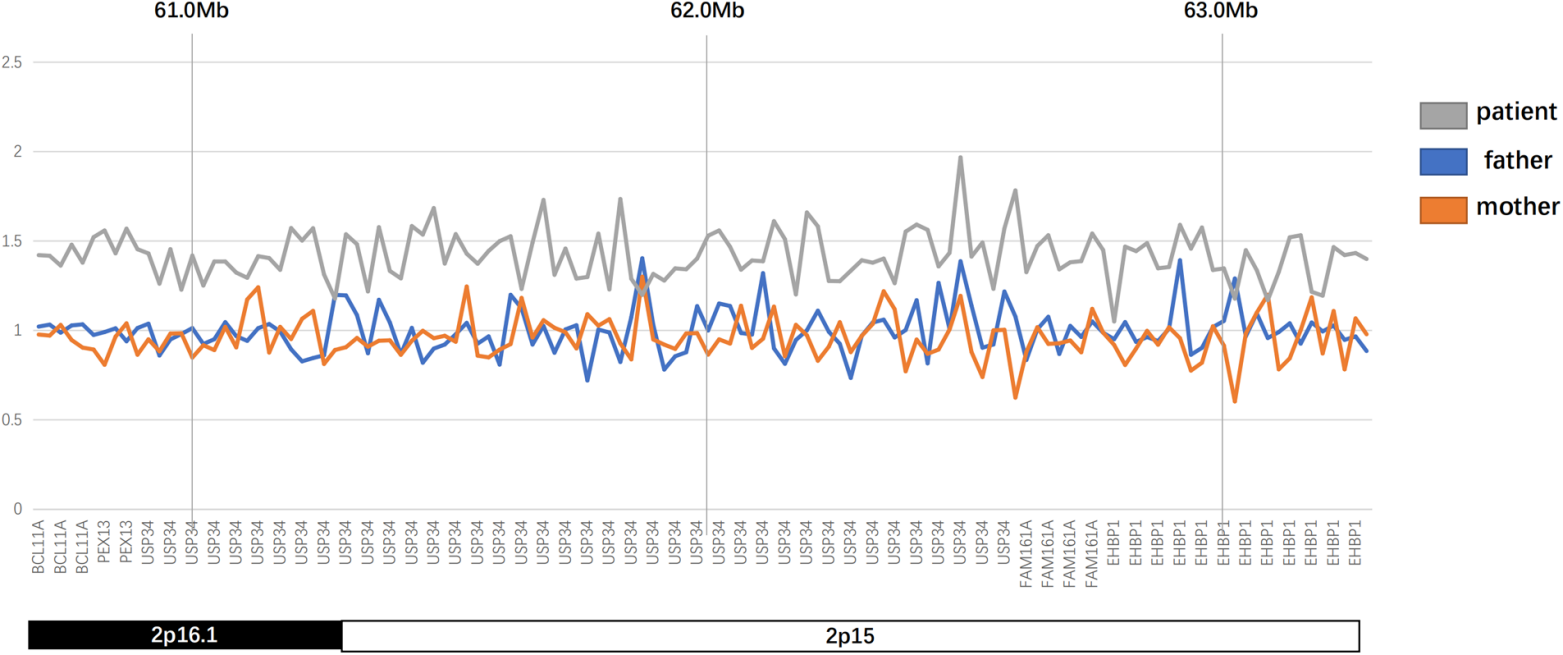
Results of CNV analysis in the patient.

**Figure 3:**
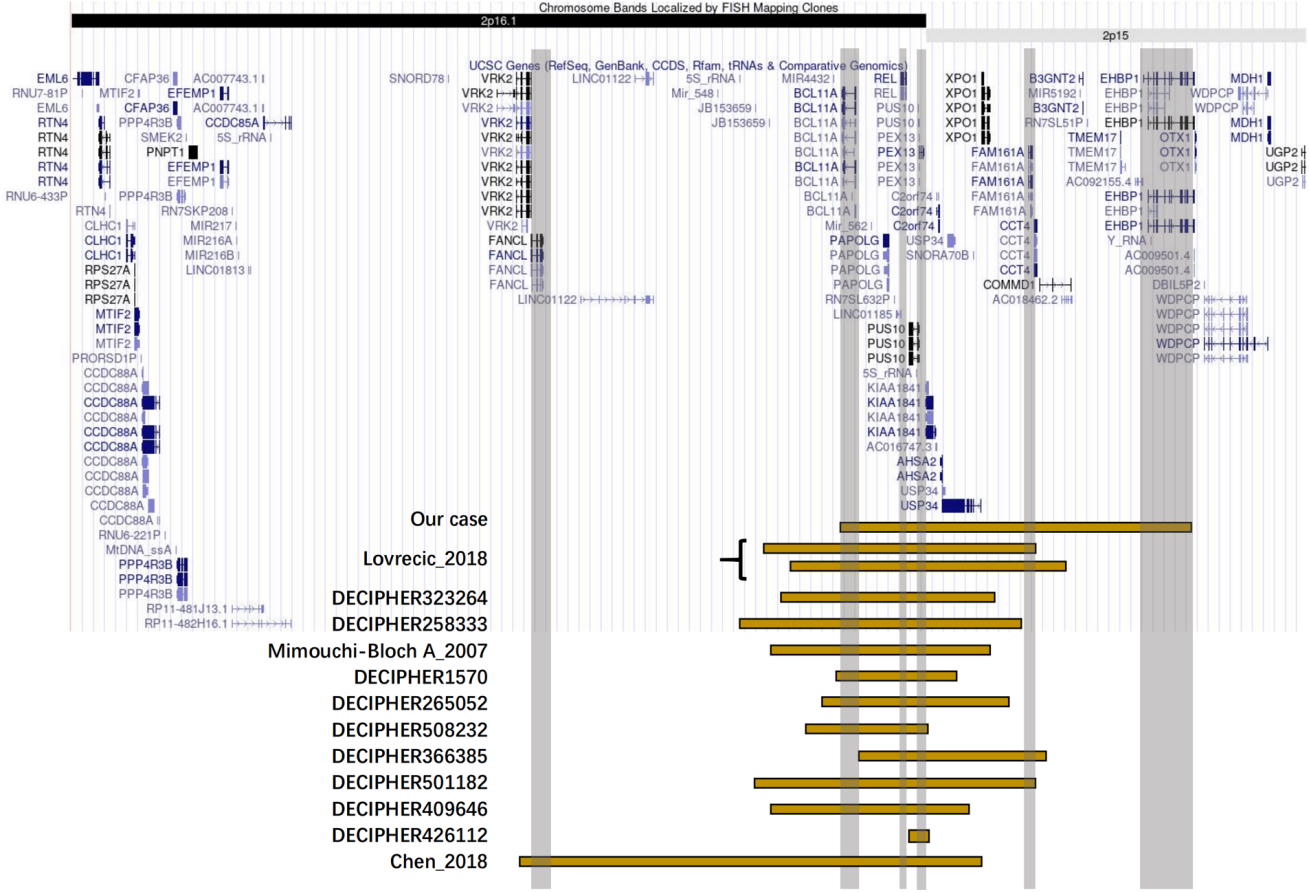
Positional plots of the microduplications on the 2p16.1p15 region.

The karyotype analysis and blood metabolism screening of the infant showed no abnormalities. Pathogenic point variants that may be associated with clinical presentation were also not detected. Further whole exome sequencing revealed that the proband had a 2.58 Mb microduplication in the paternal chromosomal 2p16.1p15 region (Figures 2, 3). We concluded that this copy number variation (CNV) may be *de novo* with the fact that sequencing data showed that neither the father nor the mother of the infant had this copy number variation. This genomic region involves multiple Online Mendelian Inheritance in Man (OMIM) genes, among which *BCL11A*, *EHBP1*, *FAM161A*, *PEX13*, and *REL* genes are identified as OMIM pathogenic genes (Supplementary Material S1). Although there were no established triplosensitive genes in this region, its phenotype is highly consistent with that of the reported cases (1, 3) (Supplementary Material S1). Using the technical standards for the interpretation and reporting of constitutional copy number variants (4), we determined the pathogenicity of chromosomal microduplication as follows. First, chromosomal 2p16.1p15 microduplication regions contain protein-coding genes (*BCL11A*, *EHBP1*, *FAM161A*, *PEX13*, and *REL*) or other known functionally important elements (score 0). Second, one breakpoint is within an established haploinsufficient gene (*BCL11A*) and the phenotype of the patient is highly specific and consistent with what is expected for the loss of function (LOF) of that gene (5–7) (score 0.45). Third, the number of protein-coding genes wholly or partially included in the copy number gain is between 0 and 34 (score 0). Then, we performed detailed assessments of genomic content using cases from the published literature, public databases, and/or in-house laboratory data. We found that the reported phenotype of *de novo* occurrences in these cases is highly specific and relatively unique to the gene or genomic region (1, 3, 7) (score 0.45). Finally, the observed copy number gain in this case is *de novo* (score 0). Therefore, the chromosomal microduplication pathogenicity of this case is likely pathogenic (score 0.90).

## Discussion

This study reports a female infant who suffered from PAH, developmental delay, ocular lesions, and bradykinesia. The PAH in this infant was discovered during right heart catheterization prior to the proposed repair of the ASD. ASD repair surgery was canceled, and routine treatment of PAH-targeted drugs, e.g., sildenafil and bosentan, was used leading to a decreased pulmonary arterial blood pressure. To clarify the possible genetic causes of PAH in the proband and promote awareness of the possibility of PAH in such children, we performed whole exome sequencing, and a 2.58 Mb microduplication in the chromosomal 2p16.1p15 region was revealed.

The chromosomal 2p16.1p15 region is prone to copy number variation. We aimed to identify the involved genes that may play key roles by collating the clinical characteristics of patients with similar copy number variants. Then, by searching the DECIPHER database (8) and the PubMed-indexed scientific literature, we included in the analysis a total of 13 cases for which phenotypes were described with four cases appearing as case reports (1–3) and another nine cases deposited in the DECIPHER database (Supplementary Table S1). The common clinical features of 14 patients with 2p16.1p15 microduplications including our report were delayed language development, developmental delay, intellectual disability, movement disorder, and special facial features. The patients reported by Mimouni-Bloch et al. (2) and DECIPHER 501182 had ADHD, whereas patients reported by DECIPHER 501182 and DECIPHER 508232 had autistic behaviors. Interestingly, three of the 14 patients with microduplications [Lovrecic et al. (3), DECIPHER 258333, and DECIPHER 508232] had macrocephaly, which was the opposite phenotype of microdeletion patients with 44% of patients showing microcephaly. Except for the patient in our case, none of the remaining 13 patients developed PH with two patients having atrial septal defect. Audiovisual impairment was present in some patients [DECIPHER426112, Mimouni-Bloch et al. (2), the patient in our case]. In terms of infections and the blood system, the patients in both our case and DECIPHER 258333 reported recurrent infections and abnormal blood counts.

Comparative analysis of OMIM genes involved in the symptoms of the patients and their microduplication regions provides an opportunity to find the key genes of specific phenotypes. The OMIM genes involved in microduplication regions in all reported cases are *VRK2*, *FANCL*, *BCL11A*, *PAPOLG*, *REL*, *PUS10*, *PEX13*, *KIAA1841*, *C2orf74*, *ASHA2*, *USP34*, *XPO1*, *FAM161A*, *CCT4*, *COMMD1*, *B3GNT2*, *TMEM17*, and *EHBP1*. Six of them, namely, *BCL11A*, *PEX13*, *REL*, *FAM161A*, *EHBP1*, and *FANCL*, are OMIM pathogenic genes. In this case report, chromosomal microduplication regions encompassed multiple genes, and five of them were identified as OMIM genes causing diseases, namely, *BCL11A*, *PEX13*, *REL*, *FAM161A*, and *EHBP1*. *BCL11A* and *PEX13* are the two most shared OMIM pathogenic genes. *REL* and *FAM161A* were involved in 13 or five cases, respectively, while *EHBP1* was only involved in our case.

*BCL11A* (OMIM 606557) encodes B-cell chronic lymphocytic leukemia (CLL)/lymphoma 11A which is a zinc finger protein and regulates gene transcription by interacting with COUP-TF protein (5). *BCL11A* is highly expressed in the human cerebral cortex, hippocampus, and cerebellum. Mutation or deletion of *BCL11A* has been shown to cause various diseases or dysfunctions. Heterozygous mutant *BCL11A* is associated with autosomal dominant Dias–Logan syndrome or intellectual developmental disability with persistent fetal hemoglobin (OMIM 617101). Soblet et al. (6) reported that *BCL11A* frameshift mutations lead to dyskinesia and hypotonia. Cai et al. (9) found *BCL11A* mutations in patients with autism and intellectual disability. Basak et al. (10) found that loss of *BCL11A* causes schizophrenia and attention deficit hyperactivity disorder. Dias et al. (7) found that *BCL11A* haploinsufficiency can lead to neurodevelopmental defects, developmental delay, and intellectual disability. Peter et al. (11) reported the association of *BCL11A* deletion with severe speech impairment. The reason for the above phenotypes may be that *BCL11A* is related to the development and growth of nerve cells. Wiegreffe et al. (5) found that *BCL11A* controls the migration of cortical projection neurons through Sema3c. Fox et al. (12) found that *BCL11A* affects the number and types of neurons by affecting the differentiation time program of neural stem cells. Kuo et al. (13) found that *BCL11A* controls axon branching and dendrite growth by regulating the expression of *DCC* and *MAP1B*. Kuo et al. (14) also found that the known X-linked intellectual disability gene *CASK* interacts with *BCL11A* to regulate axon branching and growth. All of the above are the result of *BCL11A* copy number deficiency or mutation, and the significance of *BCL11A* copy number gain remains unclear. However, the *BCL11A* knockdown model of zebra fish developed microcephaly with reduced size (15), which was in contrast to macrocephaly in three cases. Therefore, the phenotypes caused by increased or decreased *BCL11A* copy number may be opposite in some respects. Three cases reported macrocephaly, and according to the fact that the genes shared by the microduplication region in these three cases included *BCL11A*, the copy number increase of *BCL11A* may be able to explain to some extent why patients with 2p16.1p15 microduplication syndrome have intellectual disability and developmental disability and special facial features.

*PEX13* (OMIM 601789) encodes peroxisome biogenesis factor 13, which is a peroxisome membrane protein. Deletion of *PEX13* is associated with autosomal recessive disorders of peroxisome biogenesis 11A and 11B (16, 17). *REL* (OMIM 164910) encodes c-Rel, a transcription factor of the REL/NFKB family. *REL* is required for long-term synaptic plasticity and memory function in the hippocampus (18). *REL* promotes survival and apoptosis resistance in hippocampal neurons (19) and its knockout promotes a Parkinson's disease-like phenotype (20). DNA binding of NF-*κ*B/c-Rel is reduced in the substantia nigra and peripheral blood mononuclear cells of patients with Parkinson's disease (21). *FAM161A* (OMIM613596) is a microtubule-binding protein expressed throughout the photoreceptor inner segment, enriched in the photoreceptor base connecting cilia and basal body (22, 23), which interacts with C8orf37 to promote the survival of photoreceptors (24). Deletion of *FAM161A* is associated with autosomal recessive retinitis pigmentosa 28 (RP28) (23). After knocking out *Fam161a* in mice, Beryozkin et al. (25) found that the outer segmental discs of photoreceptors were disorganized in the vertical direction and that the base of the outer segments was wider and shorter than that of those in the WT mice and eventually resulted in retinal denaturation. This may be related to retinal damage in our case.

*EHBP1* (OMIM609922) may play a role in actin reorganization which links clathrin-mediated endocytosis to the actin cytoskeleton. *EHBP1* regulates vesicle trafficking by recruiting Rab8 family members and Eps15 homology domain-containing protein 1/2 (EHD1/2) (26–28). In all 14 cases, *EHBP1* was the only OMIM causative gene that was not shared and was found in children suffering from sudden PAH. Intersectin-1s (ITSN) deficiency and expression of bioactive ITSN fragments are characteristic of PAH human and animal model lung tissue (29). Bioactive ITSN fragments promote EC proliferation in PAH lungs (30). Predescu et al. (31) found that *EHBP1* was involved in the proliferative effect of EH_IRSN_ on endothelial cells by changing the subcellular localization of *EHBP1*. The unexpected occurrence of PAH based on an atrial septal defect in our case may be due to a copy number gain of the *EHBP1* gene that promotes *EHBP1* expression in endothelial cells and diverts physiological vesicle trafficking to an alternative endocytic pathway, ultimately leading to molecular phenotypes of dysfunctional pulmonary vascular endothelial cells. This requires further evidence.

Although this case report involves only one patient, it is unique in several ways. First, this patient had a longer chromosomal microduplication region length and a greater portion of the 2p15 region, in contrast to most reported 2p16.1p15 microduplication copy number changes, which mainly occurred in the 2p16.1 region. Despite this discrepancy, this patient had clinical features, such as developmental delay and movement disturbance, similar to those of patients with 2p16.1p15 microduplication carriers. A second unique feature of this case is that the father and mother of the proband had no similar copy number variation and all chromosome copy numbers were normal, suggesting that the microduplication was a *de novo* mutation. Finally, the 2p16.1p15 microduplication in the proband involved an OMIM causative gene, *EHBP1*, which was not involved in all reported case reports in the PubMed and DECIPHER databases. *EHBP1* may be related to the rapid onset of PAH in this case.

This report has limitations. First, the cause for the 2p16.1p15 microduplication of the chromosome of the patient was not explained in this report. Whole genome sequencing and further bioinformatics should be performed to explore any possible causes. Second, the reasons for PAH in this infant have not been thoroughly explored. Although *EHBP1* was suggested as the most likely cause, quantitative polymerase chain reaction and Western blot experiments on tissue or blood samples from the infant were not performed to determine the high expression of *EHBP1*. Third, subsequent *in vitro* cell experiments to verify the effect of *EHBP1* on endothelial cell proliferation phenotype should also be performed.

In conclusion, we report in detail a case of rare 2p16.1p15 microduplication syndrome involving *EHBP1*, which suggests that the occurrence of PAH should be alerted if microduplication mutations occur in the 2p16.1p15 region and involve *EHBP1*.

## Data Availability

The original contributions presented in the study are included in the article/**Supplementary Material**, further inquiries can be directed to the corresponding author.
