# Supplementary material for: Case Report: Identification of microduplication in the chromosomal 2p16.1p15 region in an infant suffering from pulmonary arterial hypertension
Source: Front Cardiovasc Med. 2023 Oct 23;10:1219480. doi: 10.3389/fcvm.2023.1219480 (PMC10626460; doi:10.3389/fcvm.2023.1219480)
Supplement: Supplementary file 1 [file Image1.pdf]

Genomes Genome Browser Tools Mirrors Downloads My Data View Help About Us

UCSC Genome Browser on Human (GRCh37/hg19)

move <<< << < > >> >>> zoom in 1.5x 3x 10x base zoom out 1.5x 3x 10x 100x

multi-region chr2:60,687,539-63,272,635 2,585,097 bp. gene, chromosome range, search terms, help pages, see exampl go examples

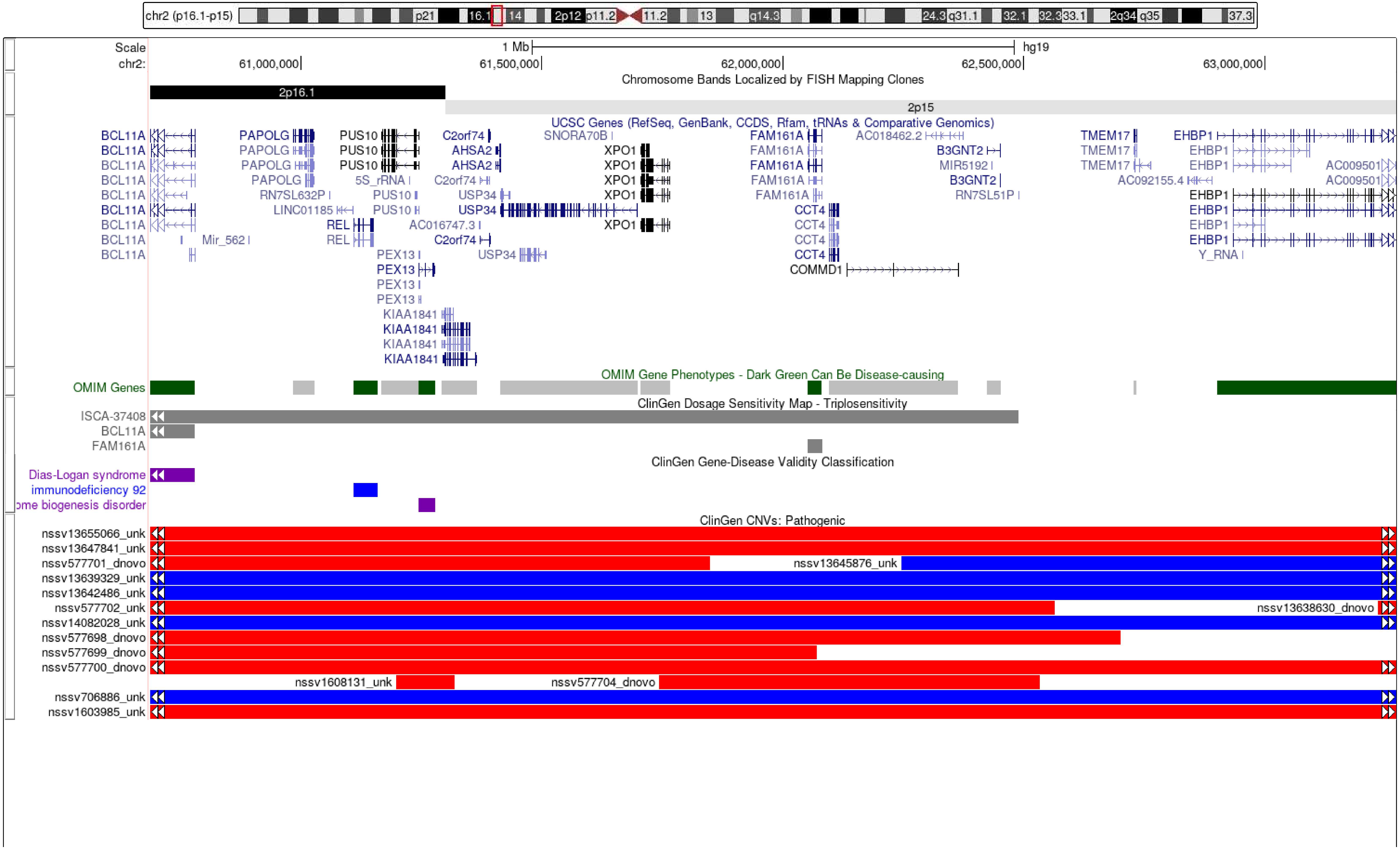

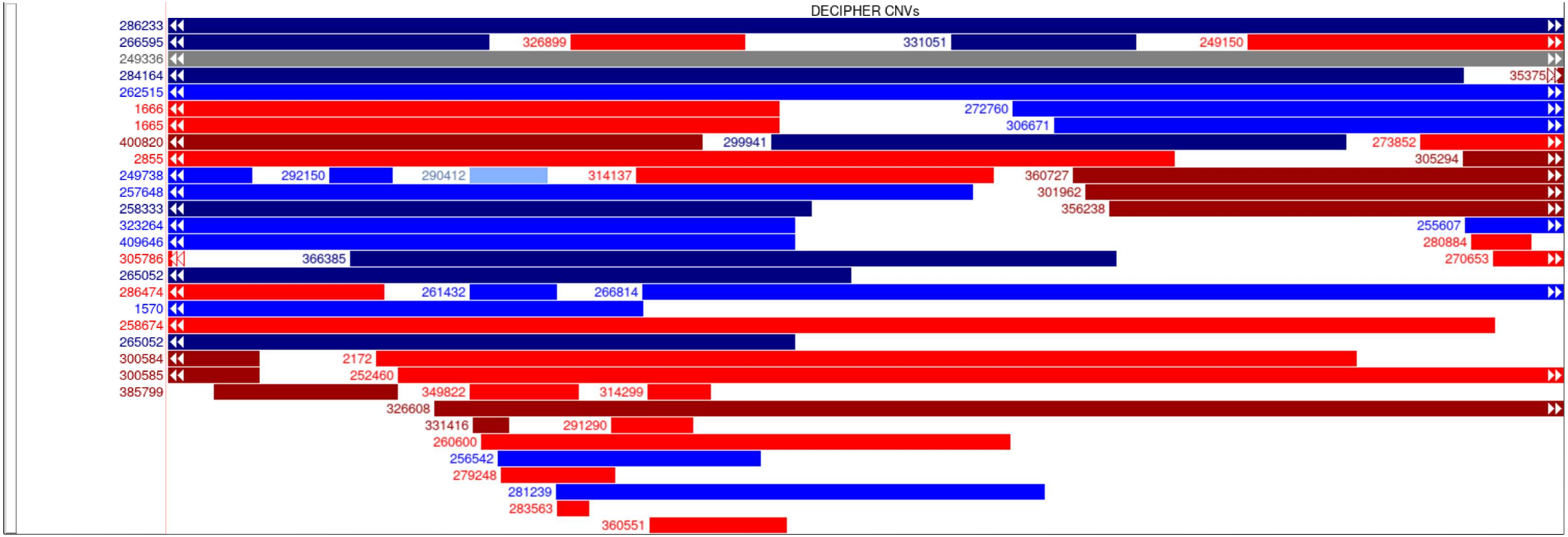

move start

< 16.0 >

Click on a feature for details. Shift+click+drag to zoom in. Click grey side bars for track options. Drag side bars or labels up or down to reorder tracks. Drag tracks left or right to new position. Press "?" for keyboard shortcuts. Use drop-down controls below and press refresh to alter tracks displayed.

move end

< 2.0 >

collapse all | track search | hide all | add custom tracks | configure | reverse | resize | expand all

Mapping and Sequencing

refresh

Base Position<br>full	P14 Fix Patches<br>hide	P14 Alt Haplotypes<br>hide	Assembly<br>hide	BAC End Pairs<br>hide	BU ORChID<br>hide	Chromosome Band<br>full	18 deCODE Recomb<br>hide
ENCODE Pilot<br>hide	Exome Probesets<br>hide	18 FISH Clones<br>hide	18 Fosmid End Pairs<br>hide	Gap<br>hide	GC Percent<br>hide	GRC Incident<br>hide	GRC Map Contigs<br>hide
Hg18 Diff<br>hide	Hg38 Diff<br>hide	Hi Seq Depth<br>hide	INSDC<br>hide	liftOver & ReMap<br>hide	LRG Regions<br>hide	Map Contigs<br>hide	Mappability<br>hide
Problematic Regions<br>hide	Recomb Rate<br>hide	RefSeq Acc<br>hide	Restr Enzymes<br>hide	Short Match<br>hide	STS Markers<br>hide		

Genes and Gene Predictions

refresh

UCSC Genes<br>pack	NCBI RefSeq<br>hide	CCDS<br>hide	CRISPR Targets<br>hide	Ensembl Genes<br>hide	17 EvoFold<br>hide	Exoniphy<br>hide	GENCODE Versions<br>hide
H-Inv 7.0<br>hide	HGNC<br>hide	IKMC Genes Mapped<br>hide	lincRNAs<br>hide	LRG Transcripts<br>hide	MGC Genes<br>hide	Old UCSC Genes<br>hide	ORFeome Clones<br>hide
Other RefSeq<br>hide	Pfam in UCSC Gene<br>hide	Prediction Archive<br>hide	Retroposed Genes<br>hide	sno/miRNA<br>hide	TransMap V5<br>hide	tRNA Genes<br>hide	UCSC Alt Events<br>hide
UniProt<br>hide	Vega Genes<br>hide	Yale Pseudo60<br>hide					

Phenotype and Literature

refresh

Publications hide | CADD hide | ClinGen full | ClinGen CNVs full | ClinVar Variants hide | Constraint scores hide | Coriell CNVs hide | COSMIC Regions hide
DECIPHER CNVs pack | DECIPHER SNVs hide | Development Delay hide | GAD View hide | GenCC hide | Gene Interactions hide | GeneReviews hide | GWAS Catalog hide
Haploinsufficiency hide | HGMD Variants hide | Lens Patents hide | LOVD Variants hide | MGI Mouse QTL hide | OMIM Alleles hide | OMIM Cyto Loci hide | OMIM Genes dense
Orphanet hide | PanelApp hide | REVEL Scores hide | RGD Human QTL hide | RGD Rat QTL hide | SNPedia hide | UniProt Variants hide | Variants in Papers hide
Web Sequences hide

COVID-19

refresh

COVID GWAS v4 hide | COVID GWAS v3 hide | Rare Harmful Vars hide

mRNA and EST

refresh

CGAP SAGE hide | Gene Bounds hide | H-Inv hide | Human ESTs hide | Human mRNAs hide | Human RNA Editing hide | Other ESTs hide | Other mRNAs hide
Poly(A) hide | PolyA-Seq hide | SIB Alt-Splicing hide | Spliced ESTs hide | UniGene hide

Expression

refresh

GTEx Gene V8 hide | Allen Brain hide | Burge RNA-seq hide | CSHL Small RNA-seq hide | ENC Exon Array hide | ENC ProtGeno hide | ENC RNA-seq hide | EPDnew Promoters hide
Affy Archive hide | GIS RNA PET hide | GNF Atlas 2 hide | GTEx Gene hide | GTEx Transcript hide | GWIPS-viz Riboseq hide | Illumina WG-6 hide | PeptideAtlas hide
qPCR Primers hide | RIKEN CAGE Loc hide | Sestan Brain hide

Regulation

refresh

ENCODE Regulation hide | CD34 DnaseI hide | CpG Islands hide | ENC Chromatin hide | ENC DNA Methyl hide | ENC DNase/FAIRE hide | ENC Histone hide | ENC RNA Binding hide
ENC TF Binding hide | New FANTOM5 hide | FSU Repli-chip hide | GeneHancer hide | Genome Segments hide | GTEx Combined eQTL hide | GTEx Tissue eQTL hide | JASPAR Transcription Factors hide
NKI Nuc Lamina hide | ORegAnno hide | Rao 2014 Hi-C hide | ReMap ChIP-seq hide | Stanf Nucleosome hide | SUNY SwitchGear hide | SwitchGear TSS hide | TFBS Conserved hide
TS miRNA Targets hide | UCSF Brain Methyl hide | UMMS Brain Hist hide | UW Repli-seq hide | Vista Enhancers hide

Comparative Genomics

refresh

Conservation hide | Cons 46-Way hide | Cons Indels MmCf hide | Evo Cpg hide | GERP hide | phastBias gBGC hide | Primate Chain/Net hide | Placental Chain/Net hide
Vertebrate Chain/Net hide | CHM13 alignments hide

Neandertal Assembly and Analysis

refresh

5% Lowest S<br>hide	Cand. Gene Flow<br>[No data-chr2]	H-C Coding Diffs<br>hide	Neandertal Methyl<br>hide	Neandertal Mito<br>[No data-chr2]	Neandertal Seq<br>hide	S SNPs<br>hide	Sel Swp Scan (S)<br>hide

− Denisova Assembly and Analysis refresh

Denisova Methyl<br>hide	Denisova Seq<br>hide	Denisova Variants<br>hide	Mod Hum Variants<br>hide	Modern Derived<br>hide

− Variation refresh

dbSNP 155<br>hide	1000G Archive<br>hide	Array Probesets<br>hide	dbSNP Archive<br>hide	dbVar Common Struct Var<br>hide	Updated DGV Struct Var<br>hide	EVS Variants<br>hide	ExAC<br>hide
Genome In a Bottle<br>hide	Genome Variants<br>hide	GIS DNA PET<br>hide	gnomAD<br>hide	HAIB Genotype<br>hide	HapMap SNPs<br>hide	HGDP Allele Freq<br>hide	Platinum Genomes<br>hide

− Repeats refresh

RepeatMasker<br>hide	Interrupted Rpts<br>hide	Microsatellite<br>hide	NumtS Sequence<br>hide	Segmental Dups<br>hide	Self Chain<br>hide	Simple Repeats<br>hide	WM + SDust<br>hide

refresh
