# Supplementary material for: Case Report: Identification of microduplication in the chromosomal 2p16.1p15 region in an infant suffering from pulmonary arterial hypertension
Source: Front Cardiovasc Med. 2023 Oct 23;10:1219480. doi: 10.3389/fcvm.2023.1219480 (PMC10626460; doi:10.3389/fcvm.2023.1219480)
Supplement: Supplementary file 2 [file Table1.docx]

**Supplementary table 1. Clinical characteristics of previously reported cases with overlapping duplications**

| Patient | Our case | Lovrecic_2018 case1 | Lovrecic_2018 case2 | DECIPHER  323264 | DECIPHER 258333 | Mimouchi-Bloch_2007 | DECIPHER 1570 | DECIPHER  265052 | DECIPHER  426112 | DECIPHER 501182 | DECIPHER  366385 | Chen_2018 | DECIPHER 508232 | DECIPHER  409646 |
| --- | --- | --- | --- | --- | --- | --- | --- | --- | --- | --- | --- | --- | --- | --- |
| Coordinates of duplications (hg19) | chr2: 60687539-  63272635 | Chr2: 60113626-62111114 | Chr2: 60308869-62368583 | Chr2: 60236241– 61,848,845 | Chr2: 59938734– 62,025,519 | Chr2: 60150427-61816209 | Chr2: 60648296– 61,568,645 | Chr2: 60541781– 61,952,880 | Chr2: 61019655-61181360 | Chr2: 60042795-62006688 | Chr2: 60798008-62218021 | Chr2: 58288588-61532538 | Chr2: 60422863-61149965 | Chr2: 60154700-61621710 |
| Gender | F, 3m | M, 3y | M, 5y | F, 15y | M | M, 5y | F, 3y | F, 7y | F, 3y | M | M | F, 22y | M, 12y | M, 1y |
| Involved OMIM morbid gene | BCL11A  REL  PEX13  FAM161A EHBP1 | BCL11A  REL  PEX13 FAM161A | BCL11A  REL  PEX13 FAM161A | BCL11A  REL  PEX13 | BCL11A  REL  PEX13 | BCL11A  REL  PEX13 | BCL11A  REL  PEX13 | BCL11A  REL  PEX13 | PEX13 | BCL11A  REL  PEX13  FAM161A | REL  PEX13 FAM161A | FANCL  BCL11A  REL  PEX13 | BCL11A  REL  PEX13 | BCL11A  REL  PEX13  - |
| Development delay | + | + | + | + | + | + | + | + | + | - | - | - | - | - |
| Speech delay | + | + | + | + | + | + | + | + | + | ­- | - | - | - | - |
| Intellectual disability | + | + | + | + | + | + | + | - | - | - | + | + | - | - |
| Autism | - | - | - | - | - | ADHD | - | - | - | +，AHDH | - | - | + | - |
| Cardiovascular | Atrial septum defect; pulmonary hypertension | - | Atrial septum defect | - | - | - | Atrial septum defect | - | - | - | - | - | - | - |
| Head, neck and limbs | Visual impairment | Receding forehead; Epicanthal folds; Pronounced philtrum and Cupid’s bow; Clinodactyly; sndactyly | Macrocephaly; Concave nasal bridge; Pronounced philtrum and Cupid’s bow; Clinodactyly | - | Macrocephaly; sall ears and hands | Puffy eyelids; Broad philturm; Visual impairment | Frontal bossing; Blepharophimosis; Upslanted palpebral fissure; Low-set ears; Micrognathia; Short stature and palm; sndactyly | - | Severe hearing impairment | Retrognathia;  Syndactyly | - | - | Macrocephaly | Cleft palate |
| Other | Recurrent infections; Abnormality of erythrocytes; hypertonia | Hypotonia | Hypotonia | Epilepsy;  obesity | Recurrent infections; Abnormality of erythrocytes; Hypotonia | - | - | Obesity; Arthritis; precocity | obsolete Psychomotor retardation | - | - | - | Multiple cafe-au-lait spots | Hypotonia |
